# One of These Things is Not Like the Other: Parents’ Experience of Family-Focused Substance Use Treatment

**DOI:** 10.1007/s10488-025-01471-w

**Published:** 2025-09-05

**Authors:** Emily A. Bosk, Sarah V. Kautz

**Affiliations:** Universisty of Colorado-Denver, The State University of New Jersey, 390 George Street, Room 713, 08901 Rutgers, New Brunswick, NJ USA

**Keywords:** Substance use treatment, Infant mental health, Child welfare system, Parental substance use, Home visiting, Trauma-Informed care, Early childhood relational health, Addiction

## Abstract

Parental substance use represents a significant source of family separation in the U.S. child welfare system. Family-focused substance use treatment programs are an innovative approach to keeping families safely together while addressing the impacts of parental substance use on children and the family system. Yet, we know very little about how families experience this new type of treatment, particularly as it relates to trauma-informed care (TIC). This qualitative study examines the experience of 24 parents participating in the In-Home Recovery Program (IHRP), an intervention that provides substance use and early child relational health treatment to families at risk of separation in the child welfare system. A content analysis indicated that IHRP was highly valued by families. The majority of participants experienced the intervention as uniquely helpful and supportive compared to other forms of substance use treatment they had previously received. Results suggest that IHRP is a model that operationalizes principles of TIC and which could be expanded as a supportive approach to parental substance use in the child welfare system to prevent family separation.

## Introduction

### Parental Substance Use and Family Separation

Approximately two million children live in a home in which their parent(s) use substances (Lipari & Van Horn, 2017). Parental substance use represents a major port of entry into the child welfare system, particularly for families with young children (defined as age 6 and under). Currently, more than a quarter of substantiated child welfare cases (in which allegations are found to be true) indicate parental substance use (U.S. Department of Health and Human Services, 2023). Child welfare cases involving parental substance use tend to have worse outcomes compared to other child welfare cases at every stage, from investigation to removal (Marsh et al., 2006). As the opioid epidemic has expanded, so too has the impact on child welfare involvement. Child removals and subsequent out-of-home placements have substantially increased for children whose parents use substances, even as removals for other reasons have declined (Meinhofer & Anglero-Diaz, 2019). Approximately 40% of children in out-of-home placements have parents who use substances (AFCARS, 2021).

Family separation can be devastating for children and families and is often experienced as systems-induced trauma (Bouza et al., 2018). When experienced as a trauma, family separation compounds the negative impacts of substance use on the family system and on all aspects of children’s growth and development (Blake et al., 2024; Doyle, 2013; Waldron et al., 2014). Infants and young children are particularly vulnerable to the negative impacts of both maltreatment and family separation because they are in a sensitive period of development, during which they are forming core schemas about themselves and the world through repeated interactions with their caregivers (Bowlby, 1969; Sroufe, 2005; Dozier et al., 2013; Zeanah et al., 2011). When relationships are disrupted and no relational or trauma intervention is provided, young children’s socio-emotional, cognitive, and physical development can be altered permanently (Dozier et al., 2013; Zeanah et al., 2011).

Placed roughly at twice the rate as older children (U.S. Department of Health and Human Services, 2023), infants and young children are disproportionately impacted by out-of-home placements for parental substance use. This high placement rate is particularly concerning because infants and young children can experience negative outcomes beyond the likelihood of removal. Specifically, children in out-of-home placements due to parental substance use disorder (SUD) are more likely to spend a longer time in care and less likely to be reunified than children placed for other reasons (Brewsaugh et al., 2023). The lack of relational permeance can disrupt positive developmental trajectories, which can have lasting effects well beyond childhood Williams-Butler et al., 2018). Interventions that reduce the need for out-of-home placement for parental substance use are critical for avoiding the secondary harms that accompany family separation, especially for young children who are in such a formative period of development, who need relational permanency, and who have already been impacted by parental substance use.

Examining how families experience interventions designed to prevent removals in the context of SUD is critical for understanding the acceptability of these interventions for those who will receive them. This study examines the experiences of parents in the In-Home Recovery Program (IHRP), an innovative a family-focused substance use treatment program for parents with young children (ages 0–6) involved in the child welfare system. Centering parents’ experiences of this intervention helps us identify whether there is alignment between the program’s intention to provide a supportive and trauma-informed approach and participants’ experience of IHRP.

### Barriers to SUD Treatment and SUD Treatment Completion

There are many barriers to receiving SUD treatment, particularly for women and parents. Research suggests that in the child welfare system, women with SUD have a different demographic profile compared to women without an SUD. Mothers with SUD are more likely to have lower levels of addiction severity, higher levels of economic instability, higher likelihood of a criminal justice referral, and very high levels of concrete service needs compared to mothers without SUD who are involved in the child welfare system (Grella et al., 2006; Marsh et al., 2006; Choi & Ryan, 2007). Mothers with SUD also tend to be younger and have more children than those without (Grella et al., 2006).

Parents with SUD have many service and material needs, and these needs often impede their ability to access treatment. Parents with SUD consistently cite lack of childcare, lack of transportation, the inability to take time off work to receive intensive treatment, and stigma as barriers to care (Milligan et al., 2011; Bakos-Block et al., 2022; Salameh et al., 2021). At the same time, parents with SUD frequently note that their desire to be good parents is one of their biggest motivations for seeking treatment (Hanson et al., 2015; Bakos-Block et al., 2022). Parents with SUD often find themselves in a catch-22, wanting to address their substance use to enhance their parenting but unable to do so because of their responsibilities as parents (Bosk et al., 2022; Redmond et al., 2020).

The challenges to accessing and staying in SUD treatment for parents are amplified by the lower rates of beginning and completing SUD treatment more generally. The 2021 National Survey on Drug Use and Health reported that approximately 94% of people with SUD did not seek or receive treatment (SAMSHA, 2023a). Even when people do seek treatment, many will not finish it, and most will need at least three months and additional episodes of care before achieving and maintaining sobriety (NIDA, 2019). A systematic review found that the average dropout rate for SUD treatment was 30% with a significant amount of variability based on the substance targeted for treatment and the treatment population (Lappan et al., 2020).

The low rates of treatment entry and completion for all people with SUD suggest that addiction treatment is often not adequately structured to meet clients’ needs (Bosk, 2017). There is likely a disconnect between how care is constructed and delivered and what clients find accessible and effective, both within the child welfare system and within SUD treatment more generally. Qualitative research on clients’ experiences in treatment can help us better identify program components that would reduce barriers to access and increase responsiveness and relevance. This knowledge is particularly important for families involved in the child welfare system, for whom resonant and effective treatment is essential to maintaining custody and care of their children.

## Trauma-Informed Care for Parental Substance Use

One potential reason why dropout rates for SUD treatment are high is that SUD treatment programs are not typically trauma-informed or trauma-specific (Flanagan et al., 2016; Bosk et al., 2019; Suchman et al., 2006). Standard care for SUD often focuses on behavioral interventions that may not align with the principles of trauma-informed care (TIC) and in turn, the needs of clients (Flanagan et al., 2016; Suchman et al., 2004, 2006). The lack of trauma-informed or trauma-specific interventions for SUD is problematic because trauma often underlies substance use and can be a consequence of use itself (Suchman et al., 2004, 2006; Flykt et al., 2012; Bosk et al., 2019, 2022; Van Scoyoc et al., 2015; Paris et al., 2018). The majority of adults in treatment for SUD report a trauma history (SAMSHA, 2014): of clients in addiction treatment, approximately 65% experienced maltreatment in childhood, and at least 90% reported experiencing at least one trauma in their lifetime (SAMSHA, 2014).

Trauma can also play a significant role in the development of a SUD. The more adverse childhood experiences a person has, the more likely they are to develop a SUD as an adult (Broekhof et al., 2023). Substance use can be viewed as an adaptation to trauma as people use substances to get relief from painful emotions or to avoid feeling numb (Chaplin & Sinha, 2013). There is a robust body of literature that connects complex trauma, SUD, and parenting (Suchman et al., 2004, 2006; Bosk et al., 2019, 2022; Hanson et al., 2015; Rutherford et al., 2013; Flykt et al., 2012; Pajulo et al., 2012; Peacock-Chambers, 2021). Research indicates that parents with SUD can (but do not always) struggle with different types of regulation (e.g. behavioral, affective, cognitive), which in turn, may impact their parenting and the quality of their relationship with their child. The quality of a parent’s relationship with their child impacts the child’s social-emotional development (Rutherford et al., 2013; Pajulo & Kalland, 2013). Positive developmental trajectories for children are scaffolded by safe, consistent, and sensitive care, particularly in their early years (Robinson et al., 2017). Despite the many ways in which trauma, parenting, and addiction are connected, these connections often are unexplored and unacknowledged in both SUD treatment and in child welfare service plans. When complex trauma and its impact on parenting remain unaddressed, parents may experience substance reoccurrence and continued relational challenges. Further, there is a missed opportunity to interrupt the intergenerational transmission of trauma because parent-child relationships, and their link to substance use, are not attended to. A focus on the family and family relationships within SUD treatment can be both a primary prevention (for young children) as it shifts the caregiving environment and a secondary intervention (for parents) as it responds to the complex trauma that underlies their SUD.

Clients with trauma histories (particularly complex trauma histories) may struggle in SUD treatment that does not center their traumatic experiences, or which does not provide relational safety. For example, unaddressed trauma can lead to lack of treatment engagement (SAMHSA, 2014), increased risk of reoccurrence (Mefodeva et al., 2023), and increased mental health symptoms (SAMHSA, 2014). Trauma survivors may struggle with trust, safety, and emotional regulation, posing challenges to traditional SUD treatment approaches that rely on compliance and control (Brown et al., 2012, 2021; Ouimette et al., 1997, 1999; Suchman et al., 2006). In this context, it is critical to address trauma when treating addiction. Principles of TIC include (1) safety, (2) transparency and trustworthiness, (3) collaboration and mutuality, (4) peer support and mutual aid, (5) empowerment, choice, and voice, and (6) attention to cultural issues (SAMHSA, 2023b). By definition, trauma-informed and trauma-specific approaches are relational and supportive in nature.

As the prevalence and impact of trauma has become more widely appreciated, so too have calls for trauma-specific and trauma-informed interventions. Yet it is unclear whether interventions labelled “trauma-informed care” really meet the criteria for TIC (Bosk, 2023), especially those used in the criminal justice, medical, and child welfare systems. Child welfare workers must balance attempts to be supportive with child safety mandates, creating an often-unresolvable tension between the TIC imperative to give clients “choice and voice” and the external control that the child welfare system can exert over them (Armstrong & Bosk 2021, Bosk & Feely, 2020; Tiderington et al., 2021). Child welfare decisions, especially child removals, can involve measures that families do not give their consent for and which they experience as coercive and unjust (Fong, 2020).

The extent to which the child welfare system can deliver interventions that truly operationalize principles of choice, collaboration, mutuality, and empowerment while maintaining the authority to direct family actions and behaviors (including participating in treatment) is an open question. Research has demonstrated that child protection mandates can diminish attempts to be supportive and trauma-informed (Tiderington et al., 2021; Armstrong & Bosk, 2021; Wigg et al., 2018). At the same time, research has also shown that supportive interventions in child welfare are often welcomed by parents and families (Breiner et al., 2016). It is important, therefore, to examine how parents experience supportive approaches to intervention, such as IHRP, in the context of child welfare. To date, we have very little data on how clients receive these interventions and whether interventions intended to be non-punitive are actually experienced as such when delivered via the child welfare system. This research investigates how parents enrolled in IHRP felt about the program and their care. In particular, we explore (1) whether parents experienced IHRP to be a non-punitive, supportive intervention; (2) how parents perceived the utility of the parenting component of IHRP; and (3) how parents made meaning of trauma work in relation to their SUD and parenting.

## Methods

### The In-Home Recovery Program Intervention (IHRP)

IHRP is a state adaptation based on the Family-Based Recovery model (Hanson et al., 2015, 2019). IHRP is a multi-generation home visiting program designed to provide substance use and parent-child treatment for families involved in the child welfare system. IHRP has three primary goals: (1) to help parents achieve and maintain sobriety, (2) to keep families safely together, and (3) to improve parent-child relationships. Two distinct teams make up IHRP: 1) the treatment team and the child welfare team. The treatment team is composed of a master-level substance use clinician, a master-level parent-child clinician, and a high school or bachelor-level family support specialist. The treatment team is responsible for delivering all clinical care to the client. The state contracts with a private behavioral health agency to provide IHRP clinical services. The child welfare team is composed of a child protective service worker and their supervisor. The child welfare team is responsible for ensuring that the child is safe to remain in the home for the duration of IHRP and for determining parental compliance with the service plan (the steps a parent must take to successfully close their child welfare case). The treatment team and the child welfare team are important partners in delivering IHRP. They collaborate to coordinate care when there is a substance reoccurrence, provide trauma-informed care, and hold monthly case conferences that also include supervisors to discuss case and cross team dynamics.

IHRP treatment can last up to 12 months. For the first six months, families receive three sessions in the home (two for substance use and one dyadic parent-child session). Parents can choose to engage in social club, a form of group therapy, once they have completed initial assessments and achieved sobriety. After six months, sessions decrease gradually to once per week at the time of discharge. Clinical treatment is provided face-to-face by the clinical team (not the child welfare system). One IHRP visit by the treatment team can serve as a replacement for one face-to-face visit by the child welfare worker each month.

## IHRP Pilot

IHRP began a pilot in January 2020 in a northeastern state with two treatment teams, each serving 12 families (24 families total). The purpose of the pilot was to establish the feasibility of IHRP, to evaluate the effectiveness of the program, consider implementation challenges, and engage in a continuous quality improvement process with child welfare staff and clinical team related to model fidelity and service quality. One county was selected to be the site because of higher rates of opioid use and the attendant associated morbidity and mortality among its population relative to the rest of the state. The county selected was also considered to have less accessible services for mental health and substance use treatment compared to other counties.

Prior to the start of the pilot, the treatment team received three intensive days of training in the model by the model developers. This training was supplemented with weekly reflective consultation to each team, and weekly consultation with the Director of the treatment teams. To support the evaluation of the pilot, the treatment team also received one day of training on conducting TIC assessment and on administering research protocols and procedures. The child welfare team received one day of intensive training on IHRP. Periodic additional training on the model for both the treatment and child welfare teams occurred on specific topics when leadership identified a need. Child welfare leadership, clinical leadership, the model developers, and the research team met regularly (monthly and then quarterly) to address challenges in implementation as they arose.

The pilot launched ten weeks prior to the global shutdowns in response to the COVID-19 pandemic. All IHRP leadership and staff worked quickly to adapt the intervention to this context (for a detailed discussion of these adaptations, see: Blinded, 2023). The program transitioned gradually back to in-person beginning in March of 2021 and was fully in-person beginning in 2022.

### Sampling and Recruitment

This study recruited a purposeful sample of parents who had participated in IHRP and were discharging from the program. We selected this sample so that clients could provide full accounts of their treatment experience while it was nearing completion. All participants in the pilot of IHRP who were being discharged from the program were invited to join the study. To be eligible for the study, clients needed to (1) have received treatment during the Pilot of IHRP and (2) be discharging from the program. Participants were introduced to the research through their clinical team, which emphasized the voluntary nature of the study. Eligibility requirements for a parent to participate in the IHRP pilot were: (1) have an open child welfare case, (2) have used illicit substances in the last 45-days (self-report or toxicology report), (3) have a diagnosed SUD requiring an intensive level of services, (4) be caring for a child under the age of 6 at least 50% of the time, and (5) make a commitment to not participate in any other treatment during IHRP, with the exception of Medicated Assisted Treatment (MAT). This study was approved by Rutgers University’s Institutional Review Board and reviewed by the New Jersey Department of Children and Families’ Research Review Committee.

### Data Collection

This article is part of a larger study examining the implementation and efficacy of the pilot for IHRP. Data for this smaller study included semi-structured interviews with 24 parents in the program at the end of their treatment. All interviews took place within the final six weeks of treatment to ensure that participants’ recollections were contemporaneous rather than retrospective. Interview guides were designed to elicit parents’ experiences and meaning-making of their treatment over the course of the intervention. Questions included prompts about the benefits and challenges of the program, the experience of receiving in-home care, and relationships with the child welfare and treatment team. While all IHRP clients are eligible to stay in the program for up to 12 months, the average length of stay for interviewees was just under eight and half months.

To support participants’ ability to speak freely about their experiences with treatment team dynamics, relationships with child protective services, and any difficult or upsetting experiences, interviews were conducted with a member of the research team and not with clinical or child welfare staff. Interviews ranged from 30 min to 90 min and were conducted via video conferencing software (Zoom Video Communications, 2022) or over the phone based on the parent’s preference. Interviews took place between 2020 and 2024. All interviews were audio recorded and transcribed verbatim. After identifying information was removed, the interviews were entered into NVivo12 for data management and analysis (NVivo, 2022). All quotes are presented verbatim. In some instances, quotes were edited for length, which is noted with an ellipsis. When quotes were edited for length, no meaning was changed or information omitted. Pseudonyms were assigned to all participants to protect their anonymity.

### Data Analysis

A content analysis of all interview data was utilized for this study (de Sola Pool, 1959). Under the supervision of the first author, the second author conducted an initial open coding of interviews and recorded memos (Charmaz, 2014). Examples of the first-round open codes included, “trauma”, “experience of support”, “parenting”, and “challenges to treatment”. Then the authors co-created the codebook based on initial codes. Data was then re-analyzed deductively by the second author utilizing the codebook to identify patterned regularities in the data. In a third round of coding, axial coding took place to consider relationships between codes and examine emerging themes. Any discrepancies between the first and second authors during the analytic process were resolved using consensus coding (Saldana, 2013). The analytic approach was abductive in nature moving between inductive and deductive analysis (Timmermans & Tavory 2022).

While the authors did not have a priori assumptions about what would emerge during interviews, the development of the interview protocol and subsequent analysis was guided by sensitizing concepts (Blumer, 1979) that were grounded in the authors’ clinical experience, previous work in implementation and child welfare, and knowledge of addiction. Specifically, we were alert to tensions between mandates to both support and surveil clients in child welfare, the non-linear nature of recovery, and the unique challenges of parenting young children while using substances.

This study employed several strategies for consistency and validity (Padgett, 2017). These strategies included peer debriefing, memoing, and creating an audit trail. Regular check-in meetings between authors allowed for opportunities to process any issues with data collection as well as identify any preliminary themes. The authors memoed throughout the analytic process and used negative case analysis to avoid lopsided conclusions and to explore alternative explanations (Padgett, 2017; Morrow, 2005). An audit trail was used to track decision-making for the study team.

## Results

### Demographic Characteristics

Twenty-four parents completed the discharge interview. Of those two (8%) identified as male and 22 (92%) identified as female. Twenty (84%) identified as Caucasian, one (4%) identified as African American, two (18%) identified as Hispanic or Latinx, and one (4%) identified as American Indian or Alaskan Native. Participants ranged in age from 26 to 46 years old. Among the participants, 20 (84%) completed IHRP. Of those who did not complete the program, one participant (4%) elected to end treatment at the time their DCF case closed, and 3 (12%) were referred to another form of treatment (either a higher or lower level of care). The majority of participants (*N* = 19) had previously received treatment for their SUD. Thirteen (54%) parents had been to one or more treatment programs prior to IHRP, averaging a little over two episodes of care (2.08). Four (17%) participants had attended five or more treatment programs prior to their enrollment in IHRP (Table 1).

**Table 1:** Interview sample demographics

	*n* = 24 (%)
*Gender*	
Male	2 (8)
Female	22 (92)
*Race*	
African-American	1 (4)
Caucasian	20 (84)
Hispanic or Latinx	2 (8)
American Indian or Alaskan Native	1 (4)
*Age*	
26–30	4 (17)
31–35	6 (25)
36–40	11 (46)
41–46	3 (12)
*Marital Status*	
Single	14 (59)
Domestic Partner	3 (12)
Married	5 (21)
Married and Separated	1 (4)
Widow/Widower	1 (4)
*Education Level*	
High School Complete	8 (34)
Vocational or Trade School	2 (8)
Some College	7 (30)
Two-Year College Degree	3 (12)
Four-Year College Degree	3 (12)
Graduate Work	1 (4)
*Employment*	
Unemployed	6 (25)
Stay at Home Parent	9 (37)
Part Time Employment	4 (17)
Full Time Employment	5 (21)
*Household Income*	
Unknown	1 (4)
Under $15, 000	4 (17)
$15,001 – $25,000	3 (12)
$25,001 – $40,000	5 (21)
$40,001 – $60,000	8 (34)
More than $60,001	3 (12)
*Housing Situation*	
Living with Family without Contributing to Expenses	5 (21)
Rent	11 (45)
Own	8 (34)

Seven themes emerged from analysis: (1) IHRP was a more resonant and valuable form of SUD treatment than participants had previously experienced; (2) concrete supports eased access to care and supported healing; (3) gender-specific treatment enhanced recovery by helping clients see how interpersonal violence and gender roles and norms contributed to their use; (4) the trauma-specific approach allowed parents to ‘get to the root of it’ and make connections between their trauma histories and SUD; (5) parents gained skills to enhance their emotional and behavioral regulatory capacities with regards to using substances and parenting; (6) despite the overall supportive approach of IHRP, tension with the child welfare team were a source of challenge for parents; and (7) parents found that the teaming structure of the treatment teams to be beneficial. Overall, parents reported that IHRP was a supportive intervention for themselves and their family.

### Resonant, Personalized, and Valuable

The majority of participants described IHRP as being a resonant and valuable form of treatment. Many parents noted that they were able to make connections and explore issues that they had not been able to in other forms of addiction treatment, which they attributed to the relational safety they experienced with their IHRP clinicians combined with the personalized nature of their care. As Tiffany explained:

Well so far like I said I like the approach of them coming out to the house and at home, ‘cause like I said it’s more personal and private. Whereas when I went to IOP [Intensive Outpatient], it would be a group of people just talking about their experiences and what brought them there. Sometimes a person could feel a little ashamed or not want to share as much information.

Keisha agreed:

I mean, most IOPs [Intensive Outpatient Programs] and stuff like that are a group setting, so it can obviously be a little more uncomfortable, a little bit less likely to share something that’s actually bothering you, compared to just kind of saying something because you have to, and you have to be a part of the group and vent about something. It’s nice to just feel a little more comfortable to talk to somebody one-on-one, and then … Than in a group, for me at least. I know some people probably have no problem talking in big groups and stuff like that, but it’s definitely easier. On top of that, they kind of see you’re in a situation with your house and with your kids on a personal level, so they might even just ask different things or see different things or recommend different things, just from seeing how you live and seeing the situations that they’ve seen occur with the kids and things like that. Definitely a little more personalized.

Tommy, explained how the individualized nature of the program allowed him to feel safe, which in turn enabled him to dig deeper in his treatment:

Well, at first my hope was just to get this over as quickly as possible and get these people out of my life. As time went on, I can really only stay on the surface for so long, and they kind of got to me. I was just hoping to become vulnerable, was a key. Find a little humility and get honest. I struggle with being honest, and I was just trying to form healthy relationships with people in my life, which is something I’m not very good at. I’ve seen the results based off kind of just opening up with them and revealing myself, and I’ve kind of been able to transfer over into my everyday relationships with my wife and my brother, my kids. I did get what I eventually hoped for, and I seem to continue to amass more of that as time goes on.

Anna-Beth expressed how impactful this program was by contrasting it to other therapeutic experiences in the past:

I’ve had therapists in the past and I’m just like, this woman’s not even listening to me. I’m going to her office the one day I’m sitting in the waiting room for a half hour. And it was because she didn’t see me or I don’t know, she gave some excuse. And then I have an hour session. I still only saw her for a half hour because she was late. She’s eating while we’re in the session. She’s on her phone. I don’t feel like she really gave a crap about her job…The people on this team are completely opposite. They’re there a 100%. Even for the social club parties, we did the Halloween and Christmas, they went above and beyond to make everything nice and special for everybody and all of our children…I feel like when you meet them and you start to get to know them in the first couple weeks, you can tell right off the bat that they’re there for you. They’re going to be there for you. They could tell you that. So, the cows come home, but they actually, their actions and their deliverance, they prove it.

Junia concurred, explaining that the individualized attention she got in IHRP transformed treatment from a ‘check the box’ activity to something valuable.

Yeah, I think I’ve been in IOP [Intensive Outpatient Program] three different times I would say within the last nine years and each time I’ve completed it, I never had a failed urine or anything, but I don’t know. I don’t take it seriously for some reason. I just don’t get much out of it. It’s not very personable, so when I go there, I’m just there to leave. I don’t really gain too much out of it. Sure, I always anticipate as much as I can, but sometimes even some of the IOPS, they were just not attentive to me. I would even ask for my own counselor and not even see anybody or I was like, “Okay, well, there’s that.” And then I would just go just for the attendance at that point and not care anymore and it was like that almost each time, so I was just like, “Okay.“… Yeah. My IOP experiences have been very different from the IHRP.

Junia went on to share how touched she was that one of the clinicians remembered her lost loved one’s birthday:

Well, the one thing that I can think of is back in November is my [lost loved one], her birthday. I mean, September. [Clinician] actually had remembered from a prior conversation that I had mentioned that, and she had asked if I was going to do anything. I never ever had any clinicians or anything… because I feel like they talk to so many people, so everything goes in one ear and out the other. It’s basically everybody’s story of different but the same, so it’s kind of all cliche things that get said, but she actually asked about what I was going to do, if I was going to do something special. That shocked me, like you spoke about before. That was really, really… I’m trying to think of the word, but just very kind of her. I know it’s her job to ask about these things, but also some people doing their job, they don’t ask about like, “Oh, are you doing something for her birthday?” So, it was just nice.

Parents described how the team’s individualized approach, combined with their relational skills, helped them feel safe and heard, which, in turn, led to greater therapeutic exploration.

### Concrete Supports Eased Access To Care and Supported Healing

While the in-home component of IHRP is designed for therapeutic purposes, numerous parents indicated that having the treatment team come to them helped them gain access to services that they might not have been able to utilize otherwise. Sherri explained:

I have two children. I have a six-year-old son, and I have a 21-month-old girl. And I am currently eight and a half months pregnant. So, the flexibility of them coming into the home, it was just like amazing. Because any other program, for me to have to pick up and go to an office for an hour three times a week is just kind of impossible. So, the flexibility of it was amazing. I’ve never even heard of that before. It was great.

Kendra described how IHRP helped her access treatment while also caring for her child with special needs:

No, they started out coming here and it was actually great, being that I don’t drive. My son has [diagnosis removed for privacy], so he doesn’t [inaudible] properly outside of the home too well. That was great. I thought that was just so awesome that they had come to me and I didn’t have to catch a ride or pay for a taxi and all that.

In addition to in-home services, IHRP provided other concrete supports, such as 24/7 access to care, access to a psychiatric treatment provider, and advocacy that supported parents in their recovery. Trish shared how meaningful the 24/7 care was to her:

I was surprised about, I don’t know. I didn’t expect anything because it was a new type of program, but depending on who you get, I was surprised about how they were super comforting. Me, with all the therapists I’ve been through, these two were not my friend, but more of a safety net, which I don’t feel I ever had. They were always there, they answered the phone when I called, texted me back if I was having a bad day, and that’s super important I feel like in a program.

Scaachi recalled that without being able to use the on-call line, she would have had a reoccurrence during a stressful time in her life:

I think that without their help, things became very traumatic in the last few weeks, and they were available to talk to on the phone outside of appointment times. You know what I mean? I could call [Substance Use Clinician] and say, “Hi, I really feel like drinking right now.” And she talked me through it, that kind of thing. [Parent Child Clinician] wrote me a letter to the court system because [abusive partner] found out we were being helped by DV [liaison], and [he] turned around and put a restraining order against me with false allegations. He [Parent Child Clinician] wrote me a letter to the court stating that I was in treatment and part of my treatment discussed the domestic abuse and how it impacted my sobriety.

Daisy described how beneficial having concrete supports could be for parents:

Other positives were, [the family support specialist] had a lot of resources to other things, which I already had had, basically, like with SNAP and TANF and things like that, but she always had resources, like if … God forbid … you needed a different place to stay, or housing, or anything like that. So, that’s really helpful, I would think, to someone, and it was helpful to me just knowing that if I needed anything, that person was there and was able to help me get it.

Each of these concrete supports created a holding experience for parents and worked to instill relational safety, a key component of trauma-specific interventions. Concrete supports not only facilitated access to treatment, but also helped parents maintain their recovery by responding to their crises and advocating on their behalf.

### Gender-Specific Care Enhanced Recovery

Many women recounted that the gender-specific care they received in IHRP was critical for their recovery journey, particularly in recognizing the intersection of domestic violence and SUD. Scacchi reflected:

And mind you, all during this time, I was dealing with domestic abuse from my partner. It was emotional, mental, financial, and I was so far deep in this abuse that I didn’t see it at first. As my counseling sessions went on, I started realizing how very toxic and abusive it was and how to start protecting myself.

Scaachi was able to recognize patterns of abuse that had previously been outside her awareness through her treatment. Jane offered an account of feeling shamed in mixed-gender services that made clear how difficult exploring the intersection of gender-related issues and SUD could be in other settings:

When I was in this outpatient, like two years ago, it was a huge group. It was two or three hours a night, three times a week, and it was mostly men. It was like 30 men and maybe 10 women. And I remember, I barely ever spoke, but the one time I decide to, I’m talking about my husband and money. And some guy goes, “Isn’t this something you should be talking about with your therapist?” I wasn’t talking about sex. I wasn’t talking about anything crazy. I was talking about something like … I didn’t even feel like it should have been that weird … I have to get my charger. That weird for … And I just burst into tears. I just started crying so hard, because it was so hard for me to talk, and then for this kid to just shoot me down in the middle of it. So, something like that, definitely judging or something like that, definitely I would not want somebody to do.

Velma noted that the fear of being shamed or embarrassed in a mixed-gender treatment setting kept her from participating:

And also too, a lot of places I was at it was like … I would be one of two or three girls, and there’s 40 guys. You know what I mean? I think it [IHRP] also helps too that you can talk about a lot of stuff that you wouldn’t want to talk in front of men around or a lot of people. It was perfect for me because I was always like, if I was in a program I always stayed quiet because I didn’t want to draw attention to me. You know what I mean?

Female participants in IHRP identified the personalized care as an opportunity to speak about issues that they would not otherwise explore in mixed-gender treatment, but which were a critical part of their recovery journey. IHRP’s individualized focus also allowed women participants to examine how relationships impacted their use. Male participants did not surface any content specifically related to gender and their approach to treatment in their interviews.

### Getting To the Root of It: Connecting Trauma Histories with SUD

Parents indicated that IHRP helped them recognize, face, and process trauma, particularly early childhood relational trauma. Keisha described how she found a new way of thinking about her mother through her work with her clinician:

Well with them, she actually did talk to me about my biological mom, who I do not have anything to do with. I’ve always had this one-track mind with her. She ended up dying, but I always had this one-track mind with her. She just was a horrible person because there’s 12 of us…I always thought she was selfish and then [Parent Child Clinician] just was telling me the situation with her mother. She was like, “When people go through these traumatic things, it turns them into a certain type of person. Some people, it turns them into a bad person. A lot of times, it’ll turn them into a better person or a person that could easily get run over or whatever.“… That never crossed my mind because my mom did have a very traumatic childhood and it never even crossed my mind to think, “Well, maybe this is why she did those things,” or “This is why she acted the way she acted.” That helped me get so much closure just with that exercise that we did. It really helped me get a lot of closure on it, for sure.

After helping parents recognize their trauma, clinicians would help parents connect their thoughts, feelings, and actions to their SUD. Rory shared the insight she had about her trauma and SUD in her own treatment:

Yeah, I’ve learned that [trauma] impacts me a lot. It impacts me tremendously. And [Substance Use Clinician] would constantly… Not constantly, but if I brought up something, she would circle back to the trauma. For example, the trauma that I have with my mother not being around, and the trauma that my son has with his father not being around. How it would make him feel, and then how it made me feel, and connecting all the dots about the similar traumas that we both have been through. And how it would cause me anxiety and depression and just… I don’t know what else to say. But we’ve talked about it, and I can’t put it into her words, because she said it so studiously, that I can’t recall exactly what she said. But she made me realize that my trauma has a lot to do with how I feel….

The trauma-specific nature of the program encouraged explicit work related to the intersection of trauma and substance use, which is critical for ensuring that treatment addresses root causes of SUD.

### Enhancing Emotional and Behavioral Regulation

Many parents reported that through IHRP treatment, they gained important skills to manage SUD triggers, improve communication, and enhance their parenting. Anna-Beth described how her treatment team helped her identify substance use triggers and other triggers for emotional dysregulation that she was previously unaware of:

But of course…we talked a lot also about maybe realizing what the traumas were or where they came from and how to possibly, if it is possible, how to avoid putting yourself in that situation again. So, I know a lot of my trauma, a lot of it comes from my [partner] and he was very, very abusive. So, putting myself in a situation where someone angers quickly would be a trigger. So, I know not to do that…Watch out for what could trigger you. But also, if that does happen, you’re unaware of that happening. You do wind up with an experience where something’s negative, how to cope with it. We did a lot of coping skills and different, recognizing your defense mechanisms also. But if this might have happened, this might have been how you coped with it in the past, but here are ways to cope with it in the positive in the future.

Trish explained how she was able to apply her learning about how to handle emotional triggers when her clinician shared that she was leaving.

When [Substance Use Clinician], she would take me to by the bay just to get me out of the house, and we’d do my session over there. That’s when I was told that she was leaving, and instead of, in my past, I probably would’ve cursed her off and walked home by myself or whatever. But I told her that I needed a couple minutes, I got out of the car, I smoked a cigarette, calmed myself down, and then got back in the car and we talked about it. We talked about how I’m not angry at her, I was just angry at the situation, but I felt like that was a huge step for me not acting inappropriately.

One of Ara’s treatment goals was improving communication with her co-parent, her child’s father, whom she did not live with and who could be a substance use trigger for her. Ara described how she learned in IHRP to use coping strategies to enhance her emotional and behavioral regulation.

The fact that you’re able to do it [put yourself in a timeout] I think is what’s key, because even when I’m training with my kids’ dad, I’m arguing with him. Instead of getting to that blow up level, I’m like, “All right. I’m going to take a time out.” I’m like, “I don’t want to say things. I don’t want to do things,” I know where I could go with that, so I’m just removing yourself from the situation, which just brings me back to the discussion a h*ll of a lot calmer. I think that’s a huge thing.

Cal identified during his interview how learning to recognize his internal cues and then respond to them so he could regulate his emotions and behavior:

I do a lot of praying, meditating, and just dealing with my problems in a different way. I realize that when I’m feeling stressed or I’m feeling down on a certain day, that means I’m missing something in my recovery, and I need to make it up. Or usually when I feel down, then that means I should probably go help somebody. And that’s usually one of my children, so it kind of makes it better. Fixes it.

Krista explained how external regulation facilitated her ability to be more emotionally and behaviorally regulated.

As far as, it has taught me time management, and how I do need to take time for myself, and if you feel that you’re not getting time for yourself, you need to demand it, because I have to be okay for my children at the end of the day, and if I’m not okay being myself physically and emotionally, I can’t be okay for them.” So, I do take time now out from my day. Instead of just going to the grocery store, every other week, I go and get my nails done, or sometimes I book a facial for myself, and that’s okay to do that. I’m a mom, but at the end of the day, I’m still a person too, and I need time for myself. So, yeah. I believe that they have helped me do that and understand that it’s okay to do that.

Sharon described learning to recognize her kids’ needs and experiences during IHRP treatment.

Just to be like more, I guess, attentive to my kids. That and also realizing, when I was in my substance abuse, you don’t think that you’re hurting your children. Your kids are still being bathed or still being fed. You’re still doing things with your kids, but you don’t realize how your actions really affect them until I was sober and out of that state of mind. Maybe I wasn’t directly hurting them, but indirectly I was definitely causing some issues between me and the kids. So, I feel like my relationship with my children have definitely improved.

IHRP helped parents identify, learn, and solidify coping, communication, and parenting skills that were translatable across different areas of their life, and which supported both their parenting and their recovery. During treatment, parents also had the opportunity the ways in which their substance use may have impacted their child(ren). This.

### Tension with the Child Welfare Team

Some parents (but not all) described a tense relationship with child welfare that sharply contrasted to the support they felt from the clinical team. Ariel explained how she felt her interactions with the child welfare team were extremely punitive and that her caseworker was always adding to her service plan without justification.

I got used to IHRP seeing me as a person, they knew me. Like I said, they knew me very well. They were at my house. They knew my parents, they knew my child. They were very close and attached to me. I still am very close to them, but when you talk to [child welfare] supervisors, I was just a number on a piece of paper. She never really got to understand what they were saying. She was just going off of what she saw on paper…For example, this is like a perfect example. They had sent me to a new [substance use screening place] when I moved to my dad’s, they had sent me to a new [substance use screening place], this was after four months of treatment, all negative screens, everything was always good at home. Everything always. I was on point. They had sent me to a [substance use screening place], they started doing random ones too, [child welfare] started sending me out to random places on top. That was another requirement, this is where it started getting, I thought I was going to start getting off [supervised visitation with her child] off the case and it took an extra, I’m not even bullshitting you. I think it took like an extra four months from the moment I thought I was going to get it [supervised visitation with her child] off, to them always having another thing I needed to accomplish to get off [supervised visitation with her child].

Eve reported a similar situation; she felt her child welfare worker did not take the time to get to know her and automatically took the word of her child’s father over her.

I think the most challenging was actually for me was trying to co-parent with my son’s father and the stress or the lack of communication from him and the opposite stories he would put in the mix. So, he would tell my case worker one thing that was opposite of what was going on, which was actually another really big help because my IHRP team was here all the time. They [the IHRP team] knew what was going on, they saw what was going on with the co-parenting or lack thereof. So, they were able to translate that information to the case worker. So, I think the most challenging would be just dealing with somebody [the child welfare worker] that doesn’t have a clue what’s going on in life, quite honestly….

Ginni also noted a feeling that her child welfare worker was not on her team.

The DCP&P [Department Protection and Permanency (child welfare)] worker, I feel like she’s out to get me. I don’t trust her. When she comes because I don’t trust her, but I completely trust them [the IHRP team] and sharing anything that’s on my mind with them…She [the DCP&P case worker] doesn’t have any compassion and she’s very sneaky and it’s like she says she cares, but I feel like she doesn’t and she’s just, she has to do her job and I understand that, but she’s very strange and when she comes here, I have nothing to worry about. It’s not like I’m hiding anything from her, but I still feel very uncomfortable when she comes here. I don’t like talking to her at all here, I don’t open up to her at all, which is probably not good because she’s probably the person I should be doing that to, but I don’t feel comfortable with her at all.

Many parents did not believe that the child welfare workers were positive partners in their treatment and closing their cases. Instead, they experienced them as adversaries who undermined their progress and impeded their parenting goals. Not all parents, however, had negative experiences with the child welfare team or worker. Some parents reported that their caseworkers were professional, helpful, and increased their feeling of being supported in their recovery and their desire to maintain their children. Jayme shared that:

I was fortunate enough that I was given two super awesome women. So in the beginning when they were still in the investigative aspect of it I had this girl DCP&P [Department of Child Protection and Permanency (child welfare] case worker 1 that you could tell that she was genuinely invested and wanted to do whatever she could for me and my son and his father, to make sure that we just got through this as smoothly as possible without I guess disturbing little Index Child’s schedule. Especially with the whole schooling thing…That was so great because she was just so responsive and anything I needed she pretty much got back to me right away because I had a supervision order in place. So obviously when all this happened that was the very first thing that we wanted to work on getting dropped. And she was with me the whole way through. The day that IHR was just like, “Yeah, she’s had nothing but positive results,” she was like I had the release from the supervision thing that afternoon. So that was awesome.

Some parents who initially had negative experiences with some child welfare staff had more positive experiences when they received a new child welfare worker. Ava explains:

But the last one, the one that I have now, she’s great. She helps us. She finds resources if I need them. She’s always texting to ask if I need anything, and she definitely changed my perspective of DCP&P [Department of Child Protection and Permanency (child welfare). I thought they were kid kidnappers and I was scared of them, and now I know that they’re only here to help…I think she was the most professional one that came here. She wasn’t judgmental. It was all about helping us. It wasn’t pointing fingers and saying what I did wrong, or this is what can happen. She would explain things to me. We would do our goal, and we would set goals, and then just open arms. She was there if I needed anything. I didn’t hesitate to talk to her. I was open with her. She was polite and personable. I got along with her.

The relationship with the child welfare worker was at the heart of whether a parent experienced their child welfare involvement to be a challenging part of the intervention.

### The Benefits of Teaming

One unique feature of IHRP is its teaming approach within the treatment team. Many parents appreciated this aspect of the intervention. Tyra shared her experience with this format:

Well, I have PTSD, and the team, the fact that they can remember, be on the same page. Even though it’s not just one, I see. It’s three of them I see, and they could all be on the same page, which is awesome. ‘Cause that really helps me from having to repeat myself, and that gets annoying. So, the fact that they could stay on the same page is great. I don’t know, just their kindness, understanding, making me feel like… When I feel like a piece of s**t, I’m not, and helping me to just calm down and relax. If they recognize, they can sense or realize from my body language or whatever, how I’m speaking, that I’m off. Or I’m having a bad day. So, they’re just good at picking up of what I’m putting down.

Abby described how having three team members having specialized roles was helpful for her:

And I also liked the fact that there were a couple different therapists. Clinician 4 was like my child, worked with me and my children. Clinician 6 was more involved with me. So, like I felt like I had somebody to go to for each thing. And I feel like they weren’t bombarded with all different types of things with me. But they all knew what was going on, because they all communicated so it was great.

The clinical team’s ability to stay connected with each other about their clients was apparent though the interviews. Many parents shared how they felt cared for by all team members and how it was clear that they had informed each other of what was going on with the client. Every parent interviewed was asked what they would like to change about the program and what they believe should be kept the same. Many participants had little they wanted to see done differently. Susie exemplifies this response:

Everything [should stay the same]. The [inaudible], the people coming to your house, the adaptability, the people they hired, everything really. For me it was perfect. I can’t say for everybody, but I really feel like for a lot of people like me who have a hard time talking in front of a lot of people or who get passed over easily, it would be a perfect program.

Parents who completed the program expressed a high degree of enthusiasm for most aspects of IHRP noting that it provided opportunities to address their SUD, trauma, co-occurring mental health disorders, and parenting.

## Discussion

Most parents reported that IHRP was a supportive treatment intervention that sharply contrasted with their previous experiences of SUD treatment. Clients identified a range of critical program components distinct from other programs, which they highly valued, and the total of which translated into feeling understood, supported, and capable of change. Parents described a range of experiences in IHRP and several major themes emerged from analysis. Specifically, we found that for parents: (1) IHRP was a more resonant and valuable form of SUD treatment than participants had previously experienced; (2) concrete supports eased access to care and supported healing; (3) gender-specific treatment enhanced recovery by helping clients see how interpersonal violence and gender roles and norms contributed to their use; (4) the trauma-specific approach allowed parents to ‘get to the root of it’ and make connections between their trauma histories and SUD; (5) parents gained skills to enhance their emotional and behavioral regulatory capacities with regards to using substances and parenting; (6) despite the overall supportive approach of IHRP, tension with the child welfare team were a source of challenge; and (7) parents found that the teaming structure to be beneficial.

At the heart of every IHRP program component that parents found resonant and different was the relational safety and security they experienced from their IHRP team. More important than any specific service was the opportunity for treatment to be framed around a relationship with unconditional positive regard that worked to avoid shame, stigma, and punitive responses to clients’ behaviors that could be better understood as trauma adaptations. In this way, IHRP embodied a key tenet of trauma treatment: “Recovery can take place only within the context of relationships; it cannot occur in isolation.” (Herman, 1997, p.133) The relational approach to clients underlined all other work in the program. Even concrete services that reduced barriers to access and treatment completion, such as the psychiatric treatment provider and the in-home work, were experienced by parents to be relational. Not only did these services bridge access to care, but parents described feeling seen and heard when their team came to them or made access to other services easier. In this way, providing services that parents needed (e.g., 24/7 crisis support, transportation) was experienced by parents as care, creating an emotionally corrective experience. Parents’ overwhelmingly positive response to the relational aspects of IHRP highlights the critical nature of utilizing TIC with parents both in child welfare and in SUD treatment.

This study demonstrates that intentional strategies to provide relational safety and center provider-client relationships, as well as relationships between child protective service workers and families, were highly valued by clients. While there have been many calls for TIC and theorizing about the importance of TIC in child welfare and addiction treatment, we know relatively little about whether the intentions of these approaches resonate with clients when implemented. This research affirms that relational approaches can be experienced positively by clients in meaningful ways, even when they perceive other challenges with their treatment (e.g., tensions with the child welfare team).

Clinicians in IHRP received weekly reflective supervision, which may be another crucial component for implementing IHRP and other programs looking to infuse a trauma-specific relational approach. Reflective supervision allows for a parallel process of relational security between the clinicians and the organization with the intent that this sense of safety translates into clinicians’ work with their clients (Parrott et al., 2023). When programs like IHRP commit to TIC or trauma-specific care, they also commit to relational ways of being as an organization. For clients to feel truly supported, their clinicians must also feel truly supported. It is likely that providing a consistent space for clinicians to process their feelings about these very complicated cases helped them remain non-judgmental and positive in interactions with clients (Bosk, 2023). This commitment requires training in reflective supervision for supervisors, ongoing expert technical assistance in creating a reflective organization, and an investment in time for relationship building among all members of IHRP, as well as processing conflicts. Investments in reflective and relational approaches require more material and emotional resources, vulnerability, and experience than strictly administrative supervision. Not all programs or staff may be ready to engage in this more emotionally intensive part of the work.

The importance of TIC and a relational approach in child welfare was also highlighted in parents’ descriptions of their relationships with their child welfare caseworkers. When parents experienced their caseworker to be non-judgmental, they were more likely to report a positive experience with child welfare. This was the case even when they had a safety protection plan (which required another adult to be with the parent at all times when their child(ren) were present) in place or other more invasive requirements that exerted significant external control over them. Relationships between child welfare and parents broke down when parents perceived that they were not being believed or that the caseworker viewed them negatively. This finding is consistent with the literature on procedural justice, which emphasizes that when people feel seen and heard and are given a chance to both speak and be listened to, they are more likely to accept outcomes (like Safety Protection Plans) with which they disagree (Braciszewski et al., 2018). Training child welfare workers on a relational approach to parents may be an important intervention to improve the implementation of TIC within this system. While treating parents with respect is a basic tenet of good practice and not specific to TIC, a relational approach expands on simply being respectful to intentionally constructing interactions so that parents can collaborate, are asked their opinion, and are treated with unconditional positive regard. These practices operationalize principles of collaboration, mutuality, empowerment, transparency, and trust that are core to TIC (Bosk, 2023).

Overall, parents reported more tension and struggle in their relationships with the child welfare team, compared to their treatment team. This may reflect role differences rather than the quality of relationships, as the child welfare team was tasked with exerting more external control over clients. In contrast, the treatment team was freer to maintain a supportive relationship that did not involve the potential for punitive consequences. Alternatively, TIC practices may not have been utilized by the child welfare team, and this caused the challenges that parents reported. More research is needed to understand how child welfare programs can deliver a supportive and truly TIC approach when some of the fundamental tenets of TIC (particularly client self-determination and choice) compete with child welfare safety mandates that result in child welfare workers exerting external control over clients. Even when supportive, the relationship by definition can be adversarial. It is unclear how innovative programs administered in the child welfare system can truly embody the principles of TIC families while maintaining the ability to exert external control over clients, especially in the form of child removals. (Bosk & Feely, 2020). At the same time, IHRP was only possible because of state-level funding and support. It offered more opportunities for families to maintain their children at home than practice as usual allows. More work is needed to provide innovative services within this highly complex practice context.

One unanticipated finding was that parents experienced IHRP as providing gender-specific care and that they found this type of treatment important to their healing and recovery. While IHRP is not intentionally structured to do so, its personalized framework allowed for parents to examine and recognize aspects of their trauma histories and SUD that were gendered. Nationally, approximately 50% of women who struggle with addiction also experience gender-based violence (Beijer et al., 2018). Research suggests that women feel less comfortable in mixed-gender treatment settings and need a space that facilitates exploration of the intersection of SUD and gender-specific victimization and violence (Saraiya et al., 2024; Stone et al., 2021; Yang et al., 2018). The inability to address these issues likely compromises care and leads people to terminate SUD treatment prematurely. Grella and colleagues (2008) found that treatment completion rates and treatment outcomes were better for women who participated in gender-specific treatment compared to mixed-gender treatment, even when mixed-gender programs provided childcare or were trauma-informed. IHRP participants made clear that their treatment was enhanced by the ability to examine the gendered aspects of their trauma, addiction, parenting, and life experiences. These results add further support for the need to invest in the development of gender-specific SUD treatment and treatment that examines the intersection of complex trauma, child maltreatment, gender-based violence, and addiction.

This study also highlights how important it is to provide concrete supports for parents impacted by the child welfare system who also have a SUD. This is consistent with research suggesting that this population has more service needs that must be addressed for a person to engage in treatment fully. Providing concrete support is consistent with a person-in-environment approach that recognizes higher-order clinical work on addiction, trauma, and parenting cannot take place while basic client needs are unmet.

One promising part of these results were parents’ perceptions that they could enhance their regulatory capacities across multiple domains: in parenting, in other relationships, and with their SUD through their work in IHRP. This perception speaks to the potential impact of IHRP as an integrated intervention that targets areas of the brain and behavior altered in response to complex trauma. Parents’ ability to articulate the connection between learning concrete skills and their ability to better regulate their thoughts, feelings, and behaviors suggests that focusing on increasing regulatory capacities should be an essential part of integrated SUD and parenting treatment. Further research, however, is needed to match parent perceptions of increasing regulatory capacities with observed changes in these domains.

### Limitations

This study has several limitations. First, interviews were conducted during a pilot program and do not reflect parents’ experiences in an established program. Discharge interviews skew toward parents who graduated from or completed IHRP, creating selection bias in these accounts. Many parents whose discharges were unplanned or who did not complete treatment were not responsive to requests for interviews; therefore, our qualitative accounts are only representative of parents who completed treatment. It is likely that parents who had unplanned discharges or did not successfully complete treatment would have more mixed or negative experiences in the program than those who completed or graduated from IHRP. Future research should focus on gathering accounts of parents who dropped out of treatment to better understand the dimensions behind this choice.

Interventions that focus on preventing removals are important for centering the needs of minoritized families who are disproportionately impacted by child removals. Black and Indigenous families are not only more likely to have higher rates of child welfare involvement, but they are also more likely to have higher out-of-home placements compared to White families (Putnam-Hornstein et al., 2013; Puzzanchera & Taylor, 2021). The demographics of the parents interviewed and served by the program, however, were a majority White. 79% of all parents enrolled in IHRP were White, and 80% of parents who elected to be interviewed for both admissions and discharge interviews were White. There is a need to understand the different racial, ethnic, and cultural dimensions of parents’ experiences in IHRP, which this sample cannot speak to. There may be cultural adaptations that would make IHRP more resonant and acceptable to other client populations. As the field of child welfare grapples with the legacy of colonization and systemic racism, attention to potential variations in family experiences based on race and ethnicity is an important component to understanding the utility of any new intervention. It is also equally important to ensure that communities in which the child welfare system has most impacted have easy and early access to programs that seek to keep families together. Finally, pairing parents’ experiences in the program with data on the efficacy of IHRP would enhance our understanding on the acceptability of IHRP to clients.

## Conclusion

IHRP is an innovative approach to intervening with parental substance use in the child welfare context by keeping families together, treating the complex trauma that underlies SUD, and the relational trauma that can accompany it. Parents reported largely positive experiences with IHRP, noting that they found it to be a valuable and resonant form of treatment. The relational safety established between the IHRP teams and their clients was foundational to all other program components and likely created a cascading set of positive effects. Tense relationships between parents and child welfare caseworkers could impinge on this sense of relational security. Conversely, when parents felt positive regard from their child welfare workers, they were less likely to feel that child welfare requirements were unfair. Parents identified the 24/7 crisis support and the in-home component as being important for removing barriers to care and should be considered as core parts of other addiction interventions. IHRP’s focus on linking trauma histories with SUD, managing triggers, and increasing regulatory capacities were program components that clients identified as making a difference in their healing and recovery, and are areas that could be emphasized in other programs. Overall, parents endorsed IHRP as a supportive intervention that did not feel stigmatizing or shaming and which supported their desire to parent their children as they worked towards recovery. Keeping families together is core to social justice in the context of disparate and disproportional rates of child removals for minoritized communities. IHRP offers an approach to intervention for parental substance use in child welfare that preserves families.

## Data Availability

Data will not be available for public use.
